# When a Neglected Tropical Disease Goes Global: Knowledge, Attitudes and Practices of Italian Physicians towards Monkeypox, Preliminary Results

**DOI:** 10.3390/tropicalmed7070135

**Published:** 2022-07-14

**Authors:** Matteo Riccò, Pietro Ferraro, Vincenzo Camisa, Elia Satta, Alessandro Zaniboni, Silvia Ranzieri, Antonio Baldassarre, Salvatore Zaffina, Federico Marchesi

**Affiliations:** 1 Servizio di Prevenzione e Sicurezza Negli Ambienti di Lavoro (SPSAL), AUSL-IRCCS di Reggio Emilia, Via Amendola n.2, I-42122 Reggio Emilia, Italy; 2 Occupational Medicine Unit, Direzione Sanità, Italian Railways’ Infrastructure Division, RFI SpA, I-00161 Rome, Italy; dott.pietro.ferraro@gmail.com; 3 Health Directorate, Occupational Medicine Unit, Bambino Gesù Children’s Hospital IRCCS, I-00146 Rome, Italy; vincenzo.camisa@opbg.net (V.C.); salvatore.zaffina@opbg.net (S.Z.); 4 Department of Medicine and Surgery, University of Parma, Via Gramsci, 14, I-43126 Parma, Italy; elia.satta@studenti.unipr.it (E.S.); alessandro.zaniboni@studenti.unipr.it (A.Z.); silvia.ranzieri@unipr.it (S.R.); federico.marchesi@unipr.it (F.M.); 5 Occupational Medicine Unit, Careggi University Hospital, I-50134 Florence, Italy; antonio.baldassarre@unifi.it

**Keywords:** monkeypox virus, variola, smallpox, knowledge, attitudes, practices, vaccine hesitancy

## Abstract

Monkeypox (MPX) has been regarded as a neglected tropic disease of Western and Central Africa since the early 70s. However, during May 2022, an unprecedent outbreak of MPX has involved most of European Countries, as well as North and South America. While the actual extent of this outbreak is being assessed by health authorities, we performed a pilot study on specific knowledge, attitudes, and practices (KAP) in a sample of Italian medical professionals (24–30 May 2022; 10,293 potential recipients), focusing on Occupational Physicians (OP), Public Health Professionals (PH), and General Practitioners (GP), i.e., medical professionals more likely involved in the early management of incident cases. More specifically, we inquired into their attitude on the use of variola vaccine in order to prevent MPX infection. From a total of 566 questionnaire (response rate of 5.5%), 163 participants were included in the final analyses. Knowledge status was quite unsatisfying, with substantial knowledge gaps on all aspect of MPX. In turn, analysis of risk perception suggested a substantial overlooking of MPX as a pathogen, particularly when compared to SARS-CoV-2, TB, HIV, and HBV. Overall, 58.6% of respondents were somehow favorable to implement variola vaccination in order to prevent MPX, and the main effectors of this attitude were identified in having been previously vaccinated against seasonal influenza (adjusted Odds Ratio [aOR] 6.443, 95% Confidence Interval [95%CI] 1.798–23.093), and being favorable to receive variola vaccine (aOR 21.416; 95%CI 7.290–62.914). In summary, the significant extent of knowledge gaps and the erratic risk perception, associated collectively stress the importance of appropriate information campaigns among first-line medical professionals.

## 1. Introduction

Monkeypox virus (MPX) is a complex DNA virus that belongs to the Poxviridae family, Chordopoxvirinae subfamily, genus *Orthopoxvirus* [1,2,3]. First identified in 1956 in a group of *Cynomolgus* monkeys recently imported to Singapore [4,5], unlike the related variola virus (VARV), MPX has a wide range of hosts and reservoirs in wild animals [6], and was not recognized as a human pathogen until 1970, when the virus was isolated from a patient with suspected smallpox infection in Zaire (nowadays, Democratic Republic of Congo) [7]. Since then, MPX has become endemic in Central and West Africa, and several outbreaks have been recorded [8,9]. The first human cases in the Western Hemisphere were then reported in 2003, during a small outbreak resulting from the transmission from infected pests in the United States [10]. Since then, not only incidence in endemic areas has substantially increased [8,11,12], spreading to other African countries [13,14], but sporadic, travel-related cases in non-endemic countries have progressively multiplied [6,9,15,16,17,18,19,20].

Since 7 May 2022, an unprecedented, but not unexpected, outbreak of MPX across Europe, Americas, and Australia occurring in subjects with no established travel link to endemic areas [16,21,22,23] has then worried international health authorities that MPX may eventually evolve in a global pathogen, potentially becoming endemic also in non-African countries [24,25]. By 31 May 2022, the situation still appeared as rapidly evolving, with around 300 officially notified cases in all of Western Europe that reasonably represented an undercount of the actual disease burden, and progressively, but steadily, increased to around 5949 cases reported from 33 countries and areas throughout the European Region by 8 July 2022 [21,22,24,25]. For example, the first case of MPX infection in Italy was reported by 17 May 2022; by 31 May 2022 a total of 9 cases were reported, and the overall count rapidly climbed to over 70 cases by 17 June 2022, and 233 cases by 6 July 2022 [26,27,28].

MPX is usually characterized by a lower case-fatality-ratio (CFR) than smallpox, the latter historically averaging 30% overall in unvaccinated individuals [29]. Depending on the clade, MPX lethality has been estimated between 3.6% (95% Confidence Interval [95%CI] 1.7–6.8) for the West African Clade, and 10.6% (95%CI 8.4–13.3) for the Central African one [8,9,30]. In this regard, gene sequencing studies have associated the ongoing epidemic with West African lineage of MPX [31,32], and, in fact, by 22 June 2022, only one death was officially recorded [27]. Nonetheless, World Health Organization (WHO) and European Centre for Disease Prevention and Control (ECDC) have stressed the importance of raising awareness and providing appropriate guidance for immediate recommended actions [24,25]. In fact, one of the challenges represented by the ongoing MPX epidemic is the lack of knowledge on this pathogen, particularly among healthcare workers (HCWs), that may contribute to its evolution to a global pathogen [33,34]. Therefore, while Italy is involved in the ongoing epidemic alongside other European Union countries [22,25,31,34], we sought to assess knowledge, attitudes and practices (collectively, KAP) of Italian HCWs on MPX.

More precisely, we report on three medical categories that could be reasonably be involved in the early management of incident cases: (a) General Practitioners (GP), as they may represent the first medical professionals to be referred by patients asking for consultation [19,33,35,36]; (b) Public Health Professionals (PH), i.e., the medical professionals involved in contact tracing and implementation of early preventive measures [19,21,22,25,34]; (c) Occupational physicians (OP), i.e., the medical professionals responsible for health promotion in the workplace [37,38,39], being diffusely involved in the communication of risk, participating in the information and formation of the workers. Moreover, we assessed whether sampled medical professionals were willingly or not to implement VARV vaccination in order to cope with the increasing occurrence of MPX infections, ultimately assessing their potential positive and negative effectors.

## 2. Materials and Methods

### 2.1. Study Design

A cross-sectional online survey was performed between 24 May 2022 and 31 May 2022 according to the STROBE (Strengthening the reporting of observational studies in epidemiology; see STROBE checklist as Supplementary Table S1 statement [40]), involving medical professionals participating in five closed discussion groups. In total, the groups had 10,293 unique members, but no information could be obtained regarding how many of them were active participants.

The chief researcher (MR) initially contacted the group administrators, preventively requesting their authorization to share the study invitation. The latter included a summary of the aims of the survey and a direct link to the first page of the questionnaire (Google Forms; Google LLC; Menlo Park, CA, USA), that, in turn, included the full informed consent. Participants were then asked about their consent for study participation through a specific dichotomous question (i.e., Yes vs. No). Even though all individuals receiving the questionnaire and agreeing with the participation were able to complete the survey, only respondents who accurately compiled the questionnaire and fulfilled the main inclusion criteria (i.e., being a medical professional working as OP, GP, or PH) were included in the following analyses.

The survey was anonymous, and no personal data (e.g., name, IP address, email address, or any personal information unnecessary to the survey) were requested, saved, or tracked. Even though no monetary compensation was offered to the participants, they were preventively guaranteed that at the end of the questionnaire a full explanation of all items would be provided, representing an educative opportunity on MPX.

### 2.2. Questionnaire

Questionnaire items were specifically designed for this study through an extensive literature review [1,2,3,8,9,10,11,12,13,14,15,16,17,18,19,21,22,23,30,31,33,36,41,42,43,44,45,46,47,48], and designed as if self-reported, and not externally validated questions. Their test–retest reliability was preventively assessed through a survey on 15 HCWs completing the questionnaire at two different points in time (i.e., 20 and 24 May 2020). Items whose corresponding correlation coefficient in T1 vs. T2 was >0.80 were considered “consistent”, being ultimately included in the final questionnaire that was then delivered by 24 May 2022. The present report includes all questionnaires retrieved between 24 May and 31 May 2022.

The final questionnaire is available as Supplementary File S1, and included the following sections:

1. Main demographic data: age, gender, seniority as medical professional, medical background (i.e., working as OP, PH, GP, or other medical professional); the Italian region where the professional mainly worked and lived.
2. Knowledge Test. Participants received a total of 24 statements on MPX (e.g., “Typically, one out of 3 women is affected by migraine”; TRUE). A summary score (Knowledge Score; KS) was then calculated by adding +1 to a sum score for every correct answer, whereas a wrong indication or a missing/“don’t know” answer added 0 to the sum score (potential range 0 to 24).
3. Risk perception. Participants were requested to rate the perceived severity (C^MPX^) and the perceived frequency (F^MPX^) of MPX in Italian population by means of a fully labeled 5-point Likert scale (range: from “not significant” to “very significant”). According to Yates [49], perceived risk can be defined as a function of the perceived probability of an event and its expected consequences, and a Risk Perception Score (RPS) was therefore calculated as the product of C^MPX^ and F^MPX^ (potential range 1 to 25).
4. Attitudes and Practices. The attitude towards VARV vaccine in order to prevent MPX infection was initially inquired, focusing on both the personal acceptance and the use in the general population. Both attitudes were reported in a full scale of 1 (totally disagree) to 5 (totally agree). Medical professionals were then requested to similarly rate how important they perceived the capability of the vaccine to avoid natural infection and complications, and about their willingness to pay, both as a personal expense (i.e., how much they would accept to pay for a MPX vaccine), and from a Public Health point of view (i.e., the optimal price for a MPX vaccine). Respondents were then requested to rate through a full Likert scale 1 (totally disagree) to 5 (totally agree) whether they perceived or not MPX as a likely occurrence during daily activities in the following months, whether they perceived or not MPX as potentially affecting daily working activities, and whether they were confident of not to be able to recognize a MPX case. Respondents were then asked to rate how difficult they perceived the management of different infectious diseases in the Italian settings, and more precisely: MPX, seasonal influenza virus (SIV), SARS-CoV-2, Hepatitis B virus (HBV), tuberculosis (TB), Human Immunodeficiency Virus (HIV). All of the aforementioned disorders were rated 1 (not difficult) to 10 (very difficult). Eventually, participants were requested to report whether they had received or not VARV vaccine (vaccination mandate for all Italian newborns was enforced until 1977, then suspended and eventually abolished in 1981), SIV vaccine during previous influenza season (i.e., 2021), and SARS-CoV-2 vaccine (at least 2 doses).

### 2.3. Ethical Considerations

The questionnaire and retrieved data were handled anonymously and confidentially through an anonymous, observational design. Participants were preventively briefed about the aims and design of the study before giving their consent to the survey and guaranteed that individual participants could not be identified based on the presented material. As this study plausibly caused no harm or stigma to the participants, and did not include clinical data about participants, a preliminary evaluation by an Ethical Committee was not forcibly required according to the Italian law (Italian Official Journal. 76, dated 31 March 2008).

### 2.4. Data Analysis

In order to preventively remove duplicate questionnaires from the analyses, when two or more entries were characterized by the very same year of birth, year of graduation, reported gender, region where the professional lived and worked, or reported occupational profile, only the first entry was retained and included in the analyses, whilst the other ones were removed.

Continuous variables were initially reported as average ± SD, while categorical values were reported as percent values. Cumulative scores (i.e., KS and RPS) were initially normalized as percent values, and then dichotomized as high vs. low estimates by median value. Likert scales were similarly dichotomized, with scores for “agree” and “totally agree” aggregated as “somewhat agreeing” vs. scores from “totally disagree” to “neutral” aggregated as “somewhat disagreeing”.

Continuous variables were initially tested for normal distribution (D’Agostino and Pearson omnibus normality test). Normality distribution was assumed as rejected for *p*-value < 0.10, and variables were therefore compared through Mann–Whitney or Kruskal–Wallis tests for multiple independent samples. On the other hand, when variables passed the normality check (D’Agostino and Pearson *p*-value ≥ 0.10), they were compared by means of Student’s t-test or ANOVA, where appropriate. Association between variables was similarly assessed through Pearson’s correlation coefficient or Spearman’s rank correlation coefficient, for variables either passing or not the normality test.

Distribution of categorical variable was reported by outcome variable of being favorable or not to promote VARV vaccine in the general population in order to avoid MPX infection, and initially analyzed through chi-squared test. Internal consistency of the knowledge sections was measured through calculation of the Cronbach’s alpha. Cronbach’s alpha is a measure of how closely related a set of items are as a group, being usually considered a scale of reliability. In general, a score ≥ 0.7 is considered acceptable.

All categorical variables that at univariate analysis were significantly associated (i.e., *p* < 0.05) with outcome variables were included in a multivariable model of binary logistic regression analysis in order to calculate adjusted odds ratios (adjOR) and their respective 95%CI.

All statistical analyses were performed by means of IBM SPSS Statistics 26.0 for Macintosh (IBM Corp. Armonk, NY, USA).

## 3. Results

### 3.1. Descriptive Analysis

**Figure 1:**
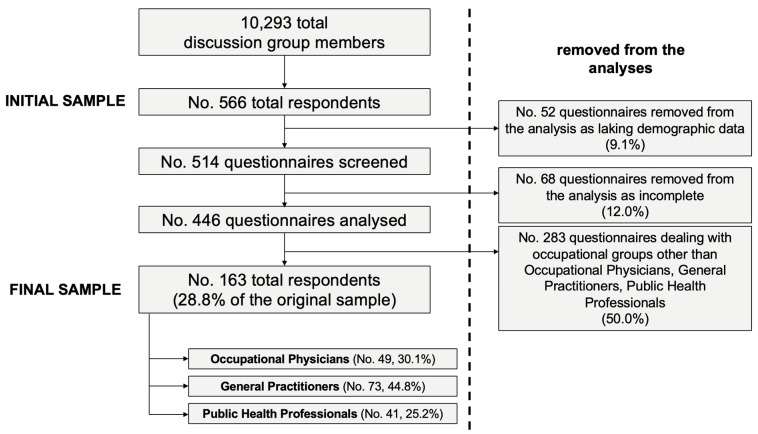
Flow char of the selection of study participants.

As shown in Figure 1, a convenience sample of 566 medical professionals (5.5% of the potentially eligible population) agreed to participating in this study. Of them, 163 fulfilled inclusion criteria, reportedly working as GP (No. = 73, 44.8%), OP (No. = 49, 30.1%), and PH (No. = 41, 25.2%).

Among the respondents included in the analyses (Table 1), the majority was female (65.0%) and the mean age 42.9 years ± 10.0 (21.5% ≥ 50-year-old), with a total seniority of 16.3 years ± 10.3 (31.3% ≥ 20 years). More than half of total respondents resided in Northern Italy (55.2%), followed by Central Italy (25.2%), and Southern Italy (17.2%). Moreover, 2.4% of participants reportedly lived and worked in another EU Country. No substantial differences were identified compared to the respondents not included in the analyses (Table A1).

### 3.2. Knowledge Test

**Figure 2:**
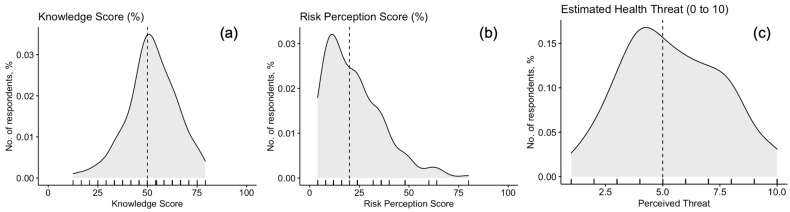
Density plots for: (**a**) Knowledge Score in 156 Italian physicians participating into the survey; (**b**) Risk Perception Score (RPS); (**c**) Perceived burden on National Health Service of Monkeypox. RPS was substantially skewed (D’Agostino–Pearson’s normality test *p*-value < 0.001), while Knowledge Score (*p* = 0.109) and perceived threat (*p* = 0.389) were not. Dotted line represents median value (50.0%, 20.0%, and 5.0, respectively).

**Figure 3:**
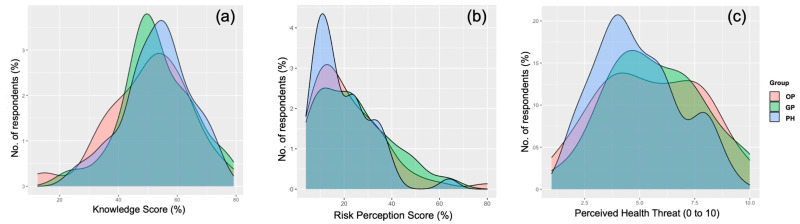
Density plots for: (**a**) Knowledge Score in 156 Italian physicians participating into the survey; (**b**) Risk Perception Score (RPS); (**c**) Perceived burden on National Health Service of Monkeypox, all of them broken down by occupational group: Occupational Physicians (OP); General Practitioners (GP); Public Health professionals (PH).

Around 27.0% of participants reportedly knew of MPX even before the inception of the current outbreak, while 42.3% had previously received any University-level formation about VARV. Nonetheless, after percent normalization, an unsatisfying KS estimate of (51.8% ± 13.0; actual range 1.0% to 79.2%, median 50.0%) was calculated. Interestingly, the distribution of the cumulative score passed the normality check (D’Agostino–Pearson normality test, *p* = 0.109) (Figure 2a), with no substantial differences between the assessed occupational groups (Figure 3a, Table A2). Nevertheless, the internal consistency coefficient amounted to Cronbach’s alpha = 0.705, suggesting an acceptable reliability of the questionnaire.

The detailed results of knowledge test are reported in Table A3. Briefly, the large majority of respondents acknowledged the MPX virus as a previously known pathogen (95.1%), and that European cases have been mostly travel-associated (82.2%). Focusing on clinical features, typical skin lesions were properly reported by 85.9% of respondents, even though most of them failed to acknowledge the prognostic significance of their number and profusion (42.9% correct answers). On the contrary, the large majority of participants acknowledged that the skin lesions may be differentially diagnosed as Varicella, Typhus, Molluscum contagiosum, Herpes simplex, and even Syphilis according to their stage (81.5%). Furthermore, the cervical and inguinal lymph nodal involvement was properly recognized by a large share of participating physicians (57.7%).

Interestingly, the majority of participants acknowledged the potential transmission of MPX through the respiratory system, by means of respiratory droplets, but also through direct contacts and body fluids (78.5%), and that standard preventive measures may be sufficient to avoid infection (74.8%). However, only 44.4% understood MPX as a pathogen circulating among various hosts, not only primates, and 42.3% had knowledge of the long survival of MPX on contaminated surfaces

The most significant uncertainty among the respondents was represented by the inappropriate understanding of actual global incidence of MPX during the last decade (i.e., around 10,000 cases: 12.3%). Even though nearly half of participants acknowledged that MPX does not evolve in uncomplicated influenza-like illness (48.5%), the high rate of systemic complications was largely overlooked (20.9%), particularly in children compared to adults (34.4%). Moreover, only 28.2% of participants were aware that the skin rash associated with MPX is typically synchronous rather than asynchronous, and even though 60.1% of respondents reportedly knew that an effective (albeit not specific) vaccine against MPX is available, with 51.2% acknowledging the availability of effective drugs, only 32.5% had knowledge that individuals previous vaccinated against VARV still require further vaccination shots. Moreover, only 17.8% of respondents were aware that smallpox has a case-fatality ratio ranging between 30% and 40%, while the majority of them (72.4%) correctly reported the lower lethality of MPX (i.e., 4% to 11%).

Eventually, two hypothetical clinical cases were presented to the participants. While a large share of respondents (77.9%) properly acknowledged a case with (a) atypical skin rash; (b) lymph nodal involvement (cervical and/or inguinal); (c) history of travel to countries endemic for MPX, as a probable MPX case, greater uncertainties were reported when facing a case characterized by generalized or localized skin rash, either maculopapular or vesiculopustular, umbilicated skin lesions, and lymphadenopathy (42.3%).

### 3.3. Risk Perception

Overall, 30.1% of participants perceived that MPX would become a likely occurrence during daily activities, and 32.5% that it could potentially affect them: when asked to rate the perceived health threat represented by MPX, an overall score of 5.42 ± 2.18 (potential range 1 to 10, actual range 1 to 10, median (5) was reported, and the correspondent distribution passed the normality check (D’Agostino–Pearson *p* = 0.389; Figure 2c) being similar among GP, PH, and OP (Figure 3c). However, only 16.6% was somewhat confident to be able to correctly recognize a MPX case.

When dealing with perceived frequency of MPX, only 3.7% of respondents acknowledged MPX infection as nowadays frequent or very frequent in European Countries, while 21.5% reportedly characterized human MPX infections as potentially severe or very severe. As a consequence, a cumulative RPS equals to 22.3% ± 14.6 was calculated (actual range: 4.0% to 80.0%, median 20.0%). The distribution of the score was visually and statistically skewed (Figure 2b, and D’Agostino–Pearson *p* < 0.001), with no substantial differences among the sampled groups of medical professionals (Figure 2b and Figure 3b; Table A1).

### 3.4. Attitudes and Practices towards MPX Vaccination

In total, 21.5% of participants reported to have been vaccinated against VARV in the past, while all of them had been vaccinated with least two doses against SARS-CoV-2 during 2021, and 84.0% was reportedly vaccinated against SIV in 2021.

Focusing on the attitude towards VARV vaccination, 64.4% would be either favorable or highly favorable to receive the vaccine, while 58.9% would acknowledge its use as instrumental in avoiding MPX infection in the general population. In this regard, the large majority of respondents rated either significant or very significant the capability of the vaccine to avoid natural infection (90.2%) and avoid systemic complications (90.8%).

When dealing with the reported willingness to pay for a candidate MPX vaccine, 31.9% of respondents were not interested, 23.9% would rate as “acceptable” a total cost < 10€ per shot, 20.2% would consider a cost of 10 to 49€ per shot, 12.9% a cost ranging from 50 to 99€ per shot, and 11.0% would agree to pay 100€ or more per shot. On the other hand, when focusing on the optimal price, the majority of respondents reported that it should be offered at no cost for the recipients (65.6%), with 16.0% recommending a cost < 10€ per shot, 14.7% acknowledging a payment between 10 and 49€ per shot, and only 3.7% of respondents agreeing for a total payment exceeding 50€ per shot (i.e., 2.5% for 50–99€, 1.2% for 100€ or more).

### 3.5. Univariate Analysis

Professionals reportedly favorable to the delivery of VARV vaccine in order to prevent MPX infections exhibited a substantially higher RPS that those against (24.4% ± 14.7 vs. 19.4% ± 14.0, *p* = 0.031), while the former scored a not significantly lower KS estimates that the latter ones (50.3% ± 14.0 vs. 54.0% ± 11.0; *p* = 0.064). In correlation analyses, not only synthetic scores were each other not correlated (rho = 0.051, *p* = 0.517), but KS was not correlated with either age (rho = 0.021; *p* = 0.787) or seniority (rho = −0.013, *p* = 0.867) of respondents, while RPS was negatively correlated with both demographic data (i.e., rho = −0.223, *p* = 0.004 for age, and rho = −0.237; *p* = 0.002 for seniority). In other words, professionals with greater age and seniority were less concerned towards MPX than the younger and less experienced ones.

**Figure 4:**
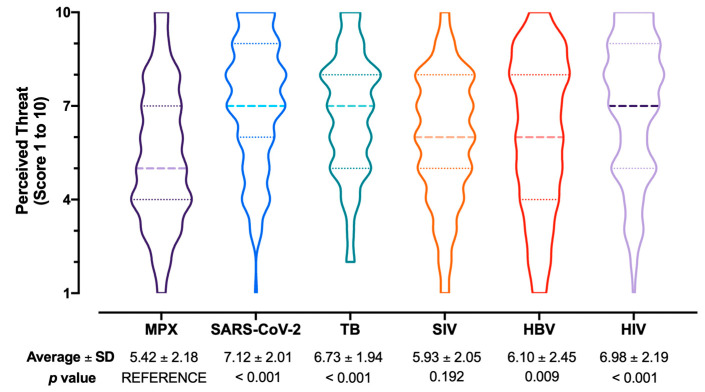
Box and violin plot for the perceived burden on National Health Service of Monkeypox (MPX) compared to SARS-CoV-2, Tuberculosis (TB), Seasonal Influenza Virus (SIV), Hepatitis B Virus (HBV), and Human Immunodeficiency Virus (HIV) infections. Comparisons were performed by mean of the Kruskal–Wallis test for multiple comparisons by assuming MPX as the reference group.

Focusing on the perceived potential health threat represented by MPX (Figure 4), it was substantially outscored by SARS-CoV-2 (score 7.12 ± 2.01; *p* < 0.001), TB (6.73 ± 1.94; *p* < 0.001), HBV (6.10 ± 2.45; *p =* 0.009), HIV (6.98 ± 2.19; *p* < 0.001), but it was similar to estimates for SIV (5.93 ± 2.05; *p* = 0.192).

In univariate analysis for dichotomous variables (Table 2), while gender, occupational group, seniority, and region of residency were not correlated with the attitude towards VARV vaccine, respondents from older age groups (i.e., age > 50 years) represented the 14.6% of those agreeing with VARV vaccination in order to prevent MPX infection, vs. 31.3% of those not agreeing (*p* = 0.018). Similarly, having a seniority as Physician > 20 years (24.0% vs. 41.8%, *p* = 0.025), having been vaccinated against VARV (13.5% vs. 32.8%, *p* = 0.006), and being not interested in paying for a putative MPX vaccine (18.8% vs. 50.7%, *p* < 0.001) were more frequently reported in professionals agreeing with VARV vaccine than in those somewhat disagreeing.

On the contrary, higher risk perception (57.3% vs. 37.3%, *p* = 0.019), a more favorable attitude towards being vaccinated (89.6% vs. 28.4%, *p* < 0.001), and having been vaccinated against SIV (93.8% vs. 70.1%, *p* < 0.001) were more frequently reported among participants favorable to the implementation of VARV vaccine against MPX than in those against.

### 3.6. Multivariable Analysis

In multivariable analysis, the outcome variable of being somewhat favorable on delivering VARV vaccine in order to prevent MPX infection was assessed through a binary logistic regression model that included the following explanatory variables: age group, seniority > 20 years, having been vaccinated against VARV, reporting higher RPS, being favorable towards being vaccinated against VARV in order to prevent MPX, having been vaccinated against SIV, and not being interested in paying for being vaccinated with VARV. Eventually, being favorable/highly favorable to receive VARV vaccination against MPX (adjOR 21.416; 95%CI 7.290 to 62.914), and having been vaccinated against SIV (adjOR 6.443; 95%CI 1.798 to 23.093) were identified as positive effectors.

## 4. Discussion

In order to properly and quickly respond to the requirements of a MPX outbreak, front-line Italian HCWs should be able to recognize and correctly manage incident cases of this previously uncommon disorder [25,33,35,36]. In turn, that requires that medical professionals have an adequate understanding of this disease, in terms of early identification, differential diagnosis, and early management of potential contacts [25,33,34]. Several medical professionals could be reasonably involved in these early response stages, most notably, GP [33]. As well as GP, OP and PH also play a critical and wholesome role in the handling of incident cases, that ranges from the contact tracing, to the implementation of preventive measures in the community (PH), and across the workplaces (OP), including vaccination campaigns [37,39,50,51]. Unfortunately, our preliminary report suggests that Italian physicians may be affected by substantial uncertainties and knowledge gaps on MPX, its clinical features, risk factors, and preventive measures. In fact, less than one fifth of them was confident in being able to properly recognize incident cases during their duties.

The reasonable explanations of these uncertainties are quite straightforward. Firstly, with a mean age of around 43 years, the majority of the respondents were born in a “Variola-free” world and did not receive a specific University-level formation on this pathogen [19,21,22,24,35,41,52]. Moreover, only one fifth of the participants declared to have heard of MPX before the ongoing outbreak: as a consequence, we cannot rule out that some of their answers to the knowledge test may have been rather based on the “common sense” than on their actual understanding of this disorder [37]. The corresponding “social desirability bias” has been acknowledged as quite common in KAP studies [37,53,54], and we cannot rule out the overstating of individuals with an effective understanding of MPX. Similarly, it is reasonable that some of their beliefs about MPX may have been collected through uncontrolled media and information sources, particularly in the first stages of the outbreak.

In fact, when dealing with MPX, clinical expertise and high suspicion index are instrumental in guaranteeing ad appropriate and timely diagnosis, as laboratory exams other than PCR may be somehow misleading. Orthopoxviruses share several common antigenic features, with a substantial cross-reactivity of elicited antibodies [11,19,42,55,56] that, in fact, represented the cornerstone of VARV vaccination [11,29,48,52,55], but still impair the reliability of most of serological exams. In settings characterized by the low suspicion index that is suggested by the very low RPS we were able to characterize, it is quite reasonable that a large share of patients may receive a proper diagnosis only in later stages of their disease, when the skin lesions have either acquired more specific features, or because of their extent and profusion [17,33,35,36,45] Interestingly enough, Adler et al. recently stressed that cases associated with the current out-of-Africa outbreak may be characterized by even more unspecific skin lesions [16], as initially reported from the USA outbreak of 2003 [10], representing a further defy for front-line professionals.

Not coincidentally, and most notably, the very same risk perception on MPX and the potential consequences of its spreading have been substantially overlooked by study respondents, even compared to similar studies performed well before the current outbreak [33,35,36]. Despite the emotional burden represented by the still ongoing SARS-CoV-2 pandemic, the emergence of a new and potentially severe pathogen was not associated with the diffuse perception of severe consequences on daily practices. Moreover, when asked to rate the potential threat represented by MPX, it was ranked by study participants well below other infectious diseases, not only including SARS-CoV-2, but also more conventional conditions such as TB, HIV, and HBV infection. Interestingly, MPX was perceived as somewhat comparable to SIV, whose actual impact on the general population has been too often overlooked [57,58,59,60,61]. KS and RPS were not correlated, thus, the latter more reasonably, therefore, results from emotional, rather than from rational, factors. This is otherwise suggested by univariate analysis, and again it appears consistent with previous studies on KAP in occupational settings [37,39]. In fact, the lack of appropriate understanding of MPX its features could, therefore, impair the appropriate implementation of preventive measures, including vaccination.

Even though the early reports on the 2022 MPX outbreak seemingly rule out that this pathogen could follow the paths of SARS-CoV-2 infection in 2020 [16,21,62], it has extensively circulated in African countries for decades, causing thousands of cases every year, with a case fatality ratio ranging from 3.6% to 11% [8,9,19,45]. In fact, MPX has been recognized as human pathogen since the 70s, and its clinical features mirror those of smallpox, with lower rates of systemic complications, and a milder outcome. Even the recently incident cases have been mostly associated with a mild course of illness [15,16], and according to the joint ECDC-WHO surveillance bulletin of 6 July 2022, no cases were reported to have died in EU/EEA countries [26,63].

The reasons of this features have been identified in some specificities of MPX. On the one hand, the conservative surface antigens guarantee some efficacy against MPX of VARV vaccines [11,36,42,52]. Despite the progressive waning of vaccine-induced immunity in older individuals, there is some evidence that the previous vaccination against VARV strongly reduces the risk to get MPX infection [45]. On the other hand, MPX, and particularly the Western-African Lineage, is characterized by deletion or substantial mutations of the main virulence factors of VARV, the agent of smallpox (i.e., the complement control protein OP-C3L, the IL-1b antagonist protein COP-C10L, and the IFN-resistance protein COP-E3L) [56,64,65]. However, human population is nowadays mostly naïve towards orthopoxviruses [8,9,11,17,30,65]; even though the reversion to a more invasive phenotype appears unlikely, this may provide the virus the opportunity to rapidly spread across susceptible individuals, causing a substantial health burden [24,25,34], that the lack of appropriate expertise of medical professionals may reasonably sharpen [22,23,25,33,35]. As a consequence, if the present outbreak does evolve into a true epidemic, the use of VARV vaccine may represent the most efficient preventive asset to be played [11,25,36,42]. Despite somewhat heterogenous indications granted by competent regulators, not only more recently authorized VARV-vaccines based on live, attenuated Vaccinia virus, Modified Vaccinia Ankara (e.g., JYNNEOS and IMVANEX), but also previously available formulates (e.g., ACAM2000), still specifically targeting VARV, have shown substantial effectiveness against MPX, that has been estimated around 85% [11,42]. Even though JYNNEOS has been approved for both VARV and MPX [42], the use of VARV vaccines against MPX would represent a sort of repurposing, and most of second generation formulates are not deprived of substantial side effect [42], this opportunity clearly should receive an appropriate and preventive health technology assessment, and will represent a significant ethical issue, similarly to those experienced during early stages of SARS-CoV-2 pandemic [66,67].

Therefore, our study not only inquired the actual knowledge status of study participants, but specifically inquired them about their acceptance of VARV vaccine as instrumental in avoid MPX infection, and the corresponding results were somewhat unexpected. First of all, even though the majority of respondents was somehow favorable towards the implementation of this policy (58.9%), neither knowledge nor risk perception were in fact associated with the attitude on vaccination. In fact, multivariable analysis identified two positive effectors, represented by having been vaccinated against SIV in the previous season (adjOR 6.443; 95%CI 1.798; 23.093), and being either favorable or highly favorable to receive VARV vaccination against MPX (adjOR 21.416; 95%CI 7.290–62.914). Again, such attitude is somewhat consistent with previous report on the KAP of HCWs towards vaccination, where a generally positive attitude towards a certain vaccine or a group of vaccines usually results in a better acceptance of new interventions (MPX, in this case) [37,50,68].

Nonetheless, a positive attitude of HCWs towards vaccines is not granted [69,70,71,72,73]. Despite their scientific background and medical training, they are often affected by a substantial degree of knowledge gaps and misbeliefs, particularly when unfamiliar with vaccinology and infectious diseases [50,74,75]. In fact, there is a certain base of evidence that even HCWs may be more strongly influenced in their attitudes towards vaccine by emotional and personal factors than by their rational understanding of this specific topic [37,50]. A low or mixed acceptance of a specific vaccine by HCWs (as in case of our survey) also represents a substantial issue from the Public Health perspective. Firstly, being that HCWs are at increased risk of acquiring and transmitting the infection to susceptible and vulnerable patients during daily health and social care tasks, they are usually priority recipients for most of vaccination programs [71,73], as during the SARS-CoV-2 pandemic [76,77,78]. In this regard, it is particularly important to stress that while SARS-CoV-2 pandemic has severely involved HCWs since its very beginning, representing a “de facto” occupational health threat, by 6 July 2022 only 15 cases of MPX have occurred in EU/EEA among HCWs, and none are known to have acquired the infection through occupational exposure [26,63].

Second, there is clear evidence that HCWs are quite effective in promoting vaccine acceptance in the general population. Unfortunately, when HCWs are themselves affected by vaccine hesitancy, they may also spread this attitude among the people they care for [69,73,76,79,80].

*Limits*. Despite its novelty and its potential significance, our study is affected by several limits. For one, in order to quickly reach a proper sample size, we opted for a web-based survey, and this design is affected by a series of implicit shortcomings, and particularly the extensive “self-selection” of participants [39,81,82]. In similarly designed studies, certain sub-groups may be unwillingly oversampled due to their greater familiarity with internet and social media, and a more proactive attitude in sharing personal information through internet and social media. Not coincidentally, even though the Italian Medical workforce has quickly aged during the last decade [83,84], the mean age of our final sample was relatively low, with a reduced share of respondents aged 50 year or older. In this regard, even though we are unable to retrieve the demographics of the participants to the original discussion group, no substantial differences were found between respondents that were included in the analyses (i.e., medical professionals working as OP, PH, and GP), and those who were otherwise excluded as reporting another medical subspecialty (Table A1). In other words, even though this subset cannot be directly compared to the overall population, we can speculate that the very same participation to an internet discussion group may have led to a preventive selection of the potentially targeted professionals, suggesting a very cautious interpretation of our results in more general terms. Moreover, it is reasonable that participating subjects may be more familiar with the assessed topic than those not participating into the study [81], with eventual self-selection of the participants and the eventual oversampling of individuals with higher understanding of a specific theme. This potential bias has a certain significance when dealing with our results, as the overall knowledge status of the study participants was far from optimal, being potentially even more unsatisfying.

Second, our sample was based on a small, convenience study group of 163 medical professionals (i.e., all the participants fulfilling inclusion criteria that had completed the questionnaire by 31 May 2022), which could be hardly considered fully representative of the national level. In fact, as previously suggested by Harapan et al. in a similar study on Indonesian GP, a conservative assumption that 50% of sampled professionals would have a good knowledge of MPX, a target sample of 382 professionals should be preferentially achieved assuming a 5% margin of error, and a confidence interval of 95% [33,35,85]. However, having been performed in the very early stages of the pandemic, shortly before the officialization of Italian National guidelines for diagnosis and management of MPX cases (25 May 2022), the present study may be of some interest for improving our understanding of the baseline understanding of medical professionals involved in the early identification and monitoring of this potentially serious infectious disease.

Third, even though discussion groups involved in the recruitment of the study participants usually performed a preventive selection (e.g., by registering only subjects who received specific invitations by the managers, answering specific “selection” questions, etc.), that allowed to estimate cross-inscriptions and actual number of individual profiles potentially reached by the original invitation, we cannot rule out that some of the respondents did not fully adhere to our selection criteria (for example, by declaring a medical specialty they actually do not practice), with a further impairment in the representativity of the sample.

**Figure A1:**
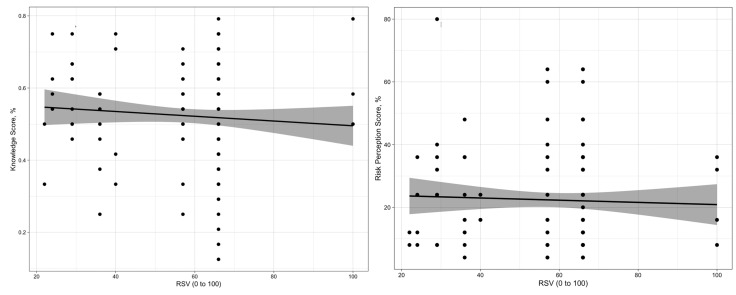
Correlation between relative search volumes (RSV) from Google Trends between 24 Mary and 31 May 2022 and Knowledge score (left; rho = −0.103, *p* = 0.189) and Risk Perception score (right; Spearman’s rho = −0.041; *p* = 0.605)).

Fourth, it should be stressed that the media coverage of MPX epidemic may have substantially influenced both the shared beliefs and the eventual risk perception of the respondents. However, no significant correlation was found between relative search volumes (RSV) for Monkeypox from Google Trends™ (i.e., the open online tool developed by Google^TM^ that reports the users’ web interest in a specific keyword through a normalized value ranging from 0 to 100, in turn proportional to the ratio between the keyword-related queries and the total of web queries) and both RPS (Spearman’s rho = −0.041; *p* = 0.605) and GKS (rho = −0.103, *p* = 0.189; Figure A1) [86]. In other words, while a proper measurement of the potential role of conventional media on the modelling of knowledge, beliefs, and perceptions remains difficult to achieve, the proxy represented by the RSV seemly suggests that new media had no substantial influence on the data reporting. On the other hand, the data we present here were collected at the beginning of the MPX epidemic, when fewer than 1/10 of Italian cases officially notified by mid-June had been identified, but greater incertitude on the potential lethality of MPX infections still existed [87]. As a consequence, our estimates should be acknowledged as strictly bounded at the exact timeframe of the study (i.e., 24–31 May 2022), and we cannot rule out that a follow up study would lead to a different outcome.

In other words, the present study was hardy generalizable, particularly in a country such as Italy, characterized by distinctive regional patterns, and considering school-specific training during the residency programs [88].

Fifth, the General Knowledge test may also require further revision and adjustments, as the early epidemiological reports suggest that the ongoing pandemic may be associated with specific clinical features and a CFR that is far lower than previously reported, even from Western African (i.e., below 0.1%) [28,63,87]. For instance, skin lesions are often inconsistently pronounced in number, size, and density, being possibly confounded with chickenpox [16,89], and cervical lymphadenopathy, that was previously acknowledged as a nearly constant clinical sign, has been reported by less than 20% of early incident cases [16,89,90], and by 27% of cases notified to ECDC by 6 July 2022 [26]. On the contrary, first reports have suggested an increased prevalence of inguinal lymph node involvement (35.3% to 48.1%) [16,89,90], anal and genital ulcers (18.5% to 57%) [89,90,91], that have been collectively explained through sexual route of transmission.

Our results should, therefore, be regarded as a pilot study, whose most significant contribution to the Public Health effort in containing the MPX outbreak is represented by the prompt availability of a rapid assessment of the actual knowledge and behavior of Italian Physicians on the eve of the first out-of-Africa MPX outbreak. On the other hand, our results should be validated and more properly defined in the following months.

## 5. Conclusions

In conclusion, despite some significant limits, our study suggests that Italian physicians potentially involved in the early management of MPX would require a more specifically tailored formation in order to guarantee their capability to cope with the requirements of their patients during the ongoing MPX outbreak. Despite the limit of the present study, and particularly the reduced sample size, our methodology could be implemented in future studies monitoring the knowledge status of HCWs towards MPX and similarly emerging pathogens.

## Figures and Tables

**Table 1:** Characteristics of the 163 Italian Physicians participating into the survey on knowledge, attitudes and practices on Monkeypox (MPX).

Variable	No./163	Average ± SD
Gender		
Male	57, 35.0%	
Female	106, 65.0%	
Age (years)		42.9 ± 10.0
Age ≥ 50 years	35, 21.5%	
Seniority (years)		16.3 ± 10.3
Seniority ≥ 20 years	51, 31.3%	
Working as …		
Occupational Physician	49, 30.1%	
General Practitioner	73, 44.8%	
Public Health Professional	41, 25.2%	
Living in …		
Northern Italy ^1^	90, 55.2%	
Central Italy ^2^	41, 25.2%	
Southern Italy/Islands ^3^	28, 17.2%	
Other EU country	4, 2.4%	
Previously vaccinated against smallpox	35, 21.5%	
Previous knowledge of MPX	44, 27.0%	
Any University-level formation on smallpox	69, 42.3%	
Acknowledging MPX infection in Europe as …		
… frequent/very frequent	6, 3.7%	
… severe/very severe	35, 21.5%	
Perceiving MPX as a likely occurrence during daily activity (agree/totally agree)	49, 30.1%	
Perceiving MPX as potentially affecting daily working activities (agree/totally agree)	53, 32.5%	
Confident to be able to recognize a MPX case (agree/totally agree)	27, 16.6%	
General Knowledge Score (%)		51.8 ± 13.0
General Knowledge Score > median (50.0%)	81, 49.7%	
Risk Perception Score		22.3 ± 14.6
Risk Perception Score > median (20.0%)	80, 49.1%	
Favorable/Highly favorable to using smallpox vaccination against MPX	96, 58.9%	
Favorable/Highly favorable to receive smallpox vaccination against MPX	105, 64.4%	
Vaccinated against SARS-CoV-2 during 2022	163, 100%	
Vaccinated against Seasonal Influenza during 2021	137, 84.0%	
Acknowledging as significant/very significant aspects for candidate MPX vaccines …		
… avoiding natural infection	147, 90.2%	
… avoiding complications	148, 90.8%	
Willingness to pay for vaccine		
Not interested	52, 31.9%	
<10€ per shot	39, 23.9%	
10–49€ per shot	33, 20.2%	
50–99€ per shot	21, 12.9%	
≥100€ per shot	18, 11.0%	
Optimal price for vaccine		
It should be offered at no cost	107, 65.6%	
<10€ per shot	26, 16.0%	
10–49€ per shot	24, 14.7%	
50–99€ per shot	4, 2.5%	
≥100€ per shot	2, 1.2%	

^1^ Aosta Valley, Piedmont, Liguria, Lombardy, Veneto, Autonomous Province of Trento, Autonomous Province of Bolzano, Friuli-Venezia-Giulia, Emilia Romagna; ^2^ Tuscany, Umbria, Marche, Lazio ^3^ Campania, Abruzzo, Apulia, Basilicata, Calabria, Sicily, Sardinia.

**Table 2:** Analysis of factors that in participating Italian physicians (No. = 163) were associated with agreeing or strongly agreeing with promoting smallpox vaccination in order to preventing Monkeypox (MPX). Comparisons were initially performed by means of chi squared test. All factors that, in univariate analysis, were associated with a favorable attitude (*p* < 0.050) were included a logistic regression analysis model as explanatory variables, with calculation of corresponding adjusted odds ratios (adjOR) and their respective 95% confidence intervals (95%CI).

Variable	Attitude towards VARV Vaccination		*p*-Value	
	Somewhat Agree (No./93, %)	Somewhat Disagree (No./63, %)		adjOR (95%CI)
Male Gender	35, 36.5%	22, 32.8%	0.756	-
Age > 50 years	14, 14.6%	21, 31.3%	0.018	2.224 (0.252; 19.645)
Seniority > 20 years	23, 24.0%	28, 41.8%	0.025	0.723 (0.176; 2.978)
Working as …			0.322	-
Occupational Physician	32, 33.3%	17, 25.4%		
General Practitioner	43, 44.8%	30, 44.8%		
Public Health Professional	21, 21.9%	20, 29.9%		
Living in …			0.401	-
Northern Italy ^1^	55, 57.3%	32, 52.2%		
Central Italy ^2^	21, 21.9%	20, 29.9%		
Southern Italy/Islands ^3^	16, 16.7%	12, 17.9%		
Other EU country	4, 4.2%	0, -		
Previously vaccinated against smallpox	13, 13.5%	22, 32.8%	0.006	0.213 (0.037; 1.223)
Previous knowledge of MPX	24, 25.0%	20, 29.9%	0.612	-
Any University-level formation on smallpox	42, 43.8%	27, 40.3%	0.781	-
Acknowledging MPX infection in Europe as …				
… frequent/very frequent	3, 3.1%	3, 4.5%	0.977	-
… severe/very severe	22, 22.9%	13, 19.4%	0.731	-
Perceiving MPX as a likely occurrence during daily activity (agree/totally agree)	31, 32.3%	18, 26.9%	0.569	
Perceiving MPX as potentially affecting daily working activities (agree/totally agree)	30, 31.3%	23, 34.3%	0.808	-
Confident to be able to recognize a MPX case (agree/totally agree)	18, 18.8%	9, 13.4%	0.494	-
Knowledge Score, > median (50.0%)	45, 46.9%	36, 53.7%	0.483	-
Risk Perception Score, > median (20.0%)	55, 57.3%	25, 37.3%	0.019	0.846 (0.348; 2.059)
Favorable/Highly favorable to receive smallpox vaccination against MPX	86, 89.6%	19, 28.4%	< 0.001	21.416 (7.290; 62.914)
Vaccinated against Seasonal Influenza during 2021	90, 93.8%	47, 70.1%	< 0.001	6.443 (1.798; 23.093)
Willingness to pay for vaccine				
Not interested to pay	18, 18.8%	34, 50.7%	< 0.001	1.047 (0.348; 3.154)
It should be offered at no cost	59, 61.5%	48, 71.6%	0.238	-

^1^ Aosta Valley, Piedmont, Liguria, Lombardy, Veneto, Autonomous Province of Trento, Autonomous Province of Bolzano, Friuli-Venezia-Giulia, Emilia Romagna; ^2^ Tuscany, Umbria, Marche, Lazio ^3^ Campania, Abruzzo, Apulia, Basilicata, Calabria, Sicily, Sardinia.

## Data Availability

The data presented in this study are available on request from the corresponding author.

## Supplementary Materials

The following are available online at https://www.mdpi.com/article/10.3390/tropicalmed7070135/s1, Table S1: STROBE Statement Checklist of items that should be included in reports of cross sectional studies, File S1: Author’s translation of the questionnaire.

## Appendix A

**Table A1:** Demographic characteristics of the respondents included (i.e., medical professionals working as Occupational Physicians, General Practitioners, or Public Health Professionals) and not included in the eventual analyses (i.e., other medical professionals) (Note: reported *p* value of Student’s t test for unpaired data in cases of continuous variables; chi squared test for categorical variables).

Variable	Respondents		*p*-Value
	Included in the Analyses (No./163, %)	Not Included in the Analyses (No./283, %)	
Male Gender	57, 35.0%	92, 32.5%	0.670
Age (years; average ± SD)	42.9 ± 10.0	42.3 ± 9.8	0.512
Seniority (years; average ± SD)	16.3 ± 10.3	15.7 ± 9.9	0.593
Living in …			0.795
Northern Italy ^1^	90, 55.2%	154, 54.4%	
Central Italy ^2^	41, 25.2%	63, 22.3%	
Southern Italy/Islands ^3^	28, 17.2%	58, 20.5%	
Other EU country	4, 2.4%	8, 2.8%	
Knowledge Score (%; average ± SD)	51.8 ± 13.9	50.9 ± 13.9	0.560
Risk Perception Score (%; average ± SD)	22.3 ± 14.6	22.6 ± 14.5	0.834
Perceived Burden (0 to 10, average ± SD)	5.4 ± 2.2	5.6 ± 2.1	0.342
Favorable/Highly favorable to using smallpox vaccination against MPX	96, 58.9%	177, 62.5%	0.446
Favorable/Highly favorable to receive smallpox vaccination against MPX	105, 64.4%	191, 64.0%	0.508

^1^ Aosta Valley, Piedmont, Liguria, Lombardy, Veneto, Autonomous Province of Trento, Autonomous Province of Bolzano, Friuli-Venezia-Giulia, Emilia Romagna; ^2^ Tuscany, Umbria, Marche, Lazio ^3^ Campania, Abruzzo, Apulia, Basilicata, Calabria, Sicily, Sardinia.

**Table A2:** Comparison of knowledge score, Risk Perception Score, Perceived Burden by occupational groups. The analyses were performed by means of Kruskal–Wallis test for multiple comparisons (the reference group was identified in Public Health Professionals).

Group	Knowledge Score (%)		Perceived Burden (0 to 10)		Risk Perception Score (%)	
	Average ± SD	*p*-Value	Average ± SD	*p*-Value	Average ± SD	*p*-Value
Occupational Physicians	50.0 ± 15.9	0.404	23.1 ± 15.5	0.509	5.6 ± 2.3	0.413
General Practitioners	52.3 ± 11.6	0.914	23.1 ± 15.2	0.464	5.5 ± 2.2	0.498
Public Health professionals	53.2 ± 11.4	REF.	20.1 ± 12.4	REF.	5.1 ± 2.0	REF.
Total	51.8 ± 13.0	-	22.3 ± 14.6	-	5.4 ± 2.2	-

**Table A3:** Knowledge test: response distribution of presented items proposed to the 163 medical professionals participating in the survey and contributing to the assessment of general knowledge score (GKS) on Monkeypox (MPX) (Cronbach’s alpha = 0.705).

Statement	Correct Answer	Total (No./163)
MPX is caused by a newly discovered virus	FALSE	154, 95.1%
MPX virus circulates only among primates, including humans	FALSE	72, 44.4%
In most cases, MPX evolves in an uncomplicated influenza-like illness	FALSE	79, 48.5%
MPX infections are associated with typical skin lesions	TRUE	140, 85.9%
Asymptomatic individuals are critical in circulating MPX	FALSE	50, 24.7%
Until recently, European cases of MPX have been mostly travel-associated	TRUE	134, 82.2%
An effective vaccine against MPX is to date available	TRUE	98, 60.1%
Effective drugs targeting MPX virus are to date available	TRUE	83, 51.2%
Recipients of VARV vaccine do not need further vaccination shots to be protected against MPX	FALSE	53, 32.5%
MPX may be transmitted …		
… through the respiratory system	FALSE	0, -
… through respiratory droplets	FALSE	2, 1.2%
… through direct contagion	FALSE	16, 9.8%
… through body fluids	FALSE	17, 10.4%
… all of the above	TRUE	128, 78.5%
Don’t know		
The case-fatality ratio of MPX usually ranges between…		
… 4% and 11%	TRUE	118, 72.4%
… 14% and 19%	FALSE	11, 6.7%
… 20% and 30%	FALSE	2, 1.2%
… 30% and 40%	FALSE	5, 3.1%
Don’t know	-	27, 16.6%
Globally, MPX in the last decade has caused around …		
… 1000 cases or less	FALSE	46, 28.4%
… 1000 to 10,000 cases	FALSE	61, 37.7%
… 10,000 cases or more	TRUE	20, 12.3%
Don’t know	-	35, 22.2%
MPX infection is associated with a high rate of systemic complications	TRUE	34, 20.9%
MPX causes a less severe illness in children (age < 14 y.o.) than in adults	FALSE	56, 34.4%
MPX infection is usually associated with a … lymphadenopathy.		
… typical, cervical and/or inguinal …	TRUE	94, 57.7%
… typical, in axillary and/or groin nodes …	FALSE	34, 20.9%
… not noticeable	FALSE	9, 5.5%
Don’t know	-	26, 16.0%
The skin rash associated with MPX is typically asynchronous	FALSE	46, 28.2%
Surface extension and profusion of MPX-associated skin lesions are of prognostic value	TRUE	70, 42.9%
MPX-associated skin lesions may be differentially diagnosed as … according to their stage		
Varicella/Varicella-Zoster	FALSE	23, 14.2%
Typhus	FALSE	2, 1.2%
Molluscum contagiosum/water warts	FALSE	5, 3.1%
Syphilis	FALSE	0, -
Herpes simplex	FALSE	0, -
All of the above	TRUE	132, 81.5%
Standard preventive measures are effective in preventing MPX infection	TRUE	122, 74.8%
A clinical case characterized by: (1) atypical skin rash; (2) lymphadenopathy (cervical and/or inguinal); (3) history of travel to countries endemic for MPX		
Confirmed MPX case	FALSE	9, 5.5%
Probable MPX case	TRUE	127, 77,9%
Doubtful MPX case	FALSE	23, 14.1%
Don’t know	-	4, 2.5%
A clinical case characterized by: (1) generalized or localized skin rash, either maculopapular or vesiculopustular; (2) umbilicated skin lesions; (3) lymphadenopathy		
Confirmed MPX case	FALSE	23, 14.1%
Probable MPX case	TRUE	69, 42.3%
Doubtful MPX case	FALSE	60, 36.8%
Don’t know	-	11, 6.7%
The case-fatality ratio of smallpox usually ranged between…		
… 4% and 11%	FALSE	47, 28.8
… 14% and 19%	FALSE	22, 13.5%
… 20% and 30%	FALSE	32, 19.6%
… 30% and 40%	TRUE	29, 17.8%
Don’t know	-	33, 20.2%
MPX is able to survive for several days on contaminated surfaces	TRUE	69, 42.3%
