# Supplementary material for: When a Neglected Tropical Disease Goes Global: Knowledge, Attitudes and Practices of Italian Physicians towards Monkeypox, Preliminary Results
Source: Trop Med Infect Dis. 2022 Jul 14;7(7):135. doi: 10.3390/tropicalmed7070135 (PMC9316880; doi:10.3390/tropicalmed7070135)
Supplement: Supplementary file 1 [file tropicalmed-07-00135-s001.zip › tropicalmed-1777096-supplementary.pdf]

Tropical Medicine and Infectious Disease

Supplementary Materials: When a Neglected Tropical Disease Goes Global: Knowledge, Attitudes and Practices of Italian Physicians towards monkeypox, Preliminary Results.

Table S1. STROBE Statement-Checklist of items that should be included in reports of cross-sectional studies.

	Item No	Recommendation	Page No
		**Title and Abstract**	
	1	(*a*) Indicate the study's design with a commonly used term in the title or the abstract	1
		(*b*) Provide in the abstract an informative and balanced summary of what was done and what was found	1
		**Introduction**	
Background/rationale	2	Explain the scientific background and rationale for the investigation being reported	1-2
Objectives	3	State specific objectives, including any prespecified hypotheses	2
		**Methods**	
Study design	4	Present key elements of study design early in the paper	2
Setting	5	Describe the setting, locations, and relevant dates, including periods of recruitment, exposure, follow-up, and data collection	2-4
Participants	6	(*a*) Give the eligibility criteria, and the sources and methods of selection of participants	2-3
Variables	7	Clearly define all outcomes, exposures, predictors, potential confounders, and effect modifiers. Give diagnostic criteria, if applicable	2-4
Data sources/ measurement	8*	For each variable of interest, give sources of data and details of methods of assessment (measurement). Describe comparability of assessment methods if there is more than one group	*2-4*
Bias	9	Describe any efforts to address potential sources of bias	2,
Study size	10	Explain how the study size was arrived at	2
Quantitative variables	11	Explain how quantitative variables were handled in the analyses. If applicable, describe which groupings were chosen and why	4
Statistical methods	12	(*a*) Describe all statistical methods, including those used to control for confounding	4
		(*b*) Describe any methods used to examine subgroups and interactions	4
		(*c*) Explain how missing data were addressed	3,4
		(*d*) If applicable, describe analytical methods taking account of sampling strategy	2
		(*e*) Describe any sensitivity analyses	-
		**Results**	
Participants	13*	(a) Report numbers of individuals at each stage of study—eg numbers potentially eligible, examined for eligibility, confirmed eligible, included in the study, completing follow-up, and analysed	5
		(b) Give reasons for non-participation at each stage	5
		(c) Consider use of a flow diagram	5
Descriptive data	14*	(a) Give characteristics of study participants (eg demographic, clinical, social) and information on exposures and potential confounders	5,6
		(b) Indicate number of participants with missing data for each variable of interest	5
Outcome data	15*	Report numbers of outcome events or summary measures	10
Main results	16	(*a*) Give unadjusted estimates and, if applicable, confounder-adjusted estimates and their precision (eg, 95% confidence interval). Make clear which confounders were adjusted for and why they were included	10
		(*b*) Report category boundaries when continuous variables were categorized	10
		(*c*) If relevant, consider translating estimates of relative risk into absolute risk for a meaningful time period	-

Other analyses	17	Report other analyses done—eg analyses of subgroups and interactions, and sensitivity analyses	8-11
**Discussion**			
Key results	18	Summarise key results with reference to study objectives	11
Limitations	19	Discuss limitations of the study, taking into account sources of potential bias or imprecision. Discuss both direction and magnitude of any potential bias	13-14
Interpretation	20	Give a cautious overall interpretation of results considering objectives, limitations, multiplicity of analyses, results from similar studies, and other relevant evidence	11-13
Generalisability	21	Discuss the generalisability (external validity) of the study results	14
**Other Information**			
Funding	22	Give the source of funding and the role of the funders for the present study and, if applicable, for the original study on which the present article is based	14

*Give information separately for exposed and unexposed group.

File S1. Author's translation of the questionnaire.

Informed Consent. Estimated colleague, the present survey has been developed and shared with the aim to assess the knowledge of medical professionals on the Monkeypox Virus. The present survey has only scientific aims. In order to thank you for your cooperation, at the end of the questionnaire (whose items are based on the available scientific evidence) the final score will be shown. Participating into the present questionnaire, you will have a chance to update your understanding of the Monkeypox Virus (by May 24, 2022).

While we thank you for your cooperation, we stress that web-based surveys must fulfill the requirements represented by the "Helsinki protocol" and EU Regulation 2016/679.

In order to fulfill the requirements of the Helsinki protocol, we're requesting to formally share your consent. Without your consent, the survey will not continue. Even after your consent, you can leave the present survey at any moment, until the sharing of the questionnaire (button "share module" at the end of the questionnaire. Moreover, we stress that the questionnaire will be registered in anonymous form, and in no way it could be associated with the compiler, as we will not retain any specific, individual information (e.g., email address, or IP address of your computer). All requested personal data are generic ones, and functional to the demographic analyses (gender, age, etc.).

According to the EU Regulation 2016/279 (GDPR), we also state that:

1) data controller, processor, as well as responsible of their retention during the analyses will be Dr. ********** whom you can ask about the process through his personal email (*********). Collected data are generic ones, with SOLE SCIENTIFIC AIMES that have been previously reported. Please be aware that all personal data must be shared with Criminal Law Authorities, without a previous personal consent, in the cases that are specifically reported by the current legal framework, without a specific request, retrieved will not be shared with third parts.
2) after the completion of the questionnaire, we cannot identify in any way the compiler; as the questionnaire is totally anonymous by design, we cannot perform any modification, correction of data collected, and their removal as well.
3) Data will be retained only for the time strictly required for the aforementioned analyses.

Do you agree to participate into the present survey? YES / NO

Section 1. At your knowledge… (please cross or circle the right statement).

	Your Answer		
MPX is caused by a newly discovered virus	True	False	Don't know
MPX virus circulates only among primates, including humans	True	False	Don't know
In most cases, MPX evolves in an uncomplicated influenza-like illness	True	False	Don't know
MPX infections are associated with typical skin lesions	True	False	Don't know
Asymptomatic individuals are critical in circulating MPX	True	False	Don't know
Until recently, European cases of MPX have been mostly travel-associated	True	False	Don't know
An effective vaccine against MPX is to date available	True	False	Don't know
Effective drugs targeting MPX virus are to date available	True	False	Don't know
Recipients of VARV vaccine do not need further vaccination shots to be protected against MPX	True	False	Don't know
MPX may be transmitted …			
… through the respiratory system		[ ]	
… through respiratory droplets		[ ]	
… through direct contagion		[ ]	
… through body fluids		[ ]	
… all of the above		[ ]	

Don’t know		[ ]	
The case-fatality ratio of MPX usually ranges between…			
… 4% and 11%		[ ]	
… 14% and 19%		[ ]	
… 20% and 30%		[ ]	
… 30% and 40%		[ ]	
Don’t know		[ ]	
Globally, MPX in the last decade has caused around …			
… 1,000 cases or less		[ ]	
… 1,000 to 10,000 cases		[ ]	
… 10,000 cases or more		[ ]	
Don’t know		[ ]	
MPX infection is associated with a high rate of systemic complications	True	False	Don’t know
MPX causes a less severe illness in children (age < 14 y.o.) than in adults	True	False	Don’t know
MPX infection is usually associated with a … lymphadenopathy.			
… typical, cervical and/or inguinal …		[ ]	
… typical, in axillary and/or groin nodes …		[ ]	
… not noticeable		[ ]	
Don’t know		[ ]	
The skin rash associated with MPX is typically asynchronous	True	False	Don’t know
Surface extension and profusion of MPX-associated skin lesions are of prognostic value	True	False	Don’t know
MPX-associated skin lesions may be differentially diagnosed as … according to their stage			
Varicella / Varicella-Zoster		[ ]	
Typhus		[ ]	
Molluscum contagiosum / water warts		[ ]	
Syphilis		[ ]	
Herpes simplex		[ ]	
All of the above		[ ]	
Standard preventive measures are effective in preventing MPX infection	True	False	Don’t know
A clinical case characterized by: (1) atypical skin rash; (2) lymphadenopathy (cervical and/or inguinal); (3) history of travel to countries endemic for MPX			
Confirmed MPX case		[ ]	
Probable MPX case		[ ]	
Doubtful MPX case		[ ]	
Don’t know		[ ]	
A clinical case characterized by: (1) generalized or localized skin rash, either maculopapular or vesiculopustular; (2) umbilicated skin lesions; (3) lymphadenopathy			
Confirmed MPX case		[ ]	
Probable MPX case		[ ]	
Doubtful MPX case		[ ]	
Don’t know		[ ]	
The case-fatality ratio of smallpox usually ranged between…			
… 4% and 11%		[ ]	
… 14% and 19%		[ ]	
… 20% and 30%		[ ]	
… 30% and 40%		[ ]	
Don’t know		[ ]	
MPX is able to survive for several days on contaminated surfaces	True	False	Don’t know

Section 2. Personal experiences with Smallpox and Monkeypox.

	**Yes**	**No**	**Don’t Remember / I Prefer Not to Answer**
At your knowledge, were you previously vaccinated against smallpox?	[ ]	[ ]	[ ]
Before 2022, did you have any knowledge of Monkeypox?	[ ]	[ ]	[ ]

During your studies, did you receive any University-level formation on Monkeypox?	[ ]	[ ]	[ ]

Section 3. Attitude towards Vaccinations.

Are you favorable to deliver Smallpox vaccine for preventing MPX infection?	
Totally disagree	[ ]
Disagree	[ ]
Neutral	[ ]
Agree	[ ]
Totally Agree	[ ]
If made available, would you receive Smallpox vaccine for preventing MPX infection?	
Totally disagree	[ ]
Disagree	[ ]
Neutral	[ ]
Agree	[ ]
Totally Agree	[ ]
How much would you pay for receiving a vaccine against Monkeypox?	
Not interested	[ ]
< 10€ per shot	[ ]
10€–49€ per shot	[ ]
50–99€ per shot	[ ]
≥ 100€ per shot	[ ]
From your point of view, what it should be the optimal price for a Monkeypox vaccine?	
It should be offered at no cost	[ ]
< 10€ per shot	[ ]
10€–49€ per shot	[ ]
50–99€ per shot	[ ]
≥ 100€ per shot	[ ]

	**Yes**	**No**	**Don't Remember / I Prefer Not to Answer**
During 2021, have you received SARS-CoV-2 vaccine? (completed basic course according to the vaccination schedule)	[ ]	[ ]	[ ]
During 2021, have you received seasonal influenza vaccine?	[ ]	[ ]	[ ]

In the design of a candidate vaccine, how would you rate the capability of …	
Avoiding natural infection	(1) Not significant at all<br>(2)<br>(3)<br>(4)<br>(5) very significant
Avoiding complications	(1) Not significant at all<br>(2)<br>(3)<br>(4)<br>(5) very significant

Section 4. Perceptions and beliefs about Monkeypox (MPX) Virus infections.

**According to your current understanding, how you perceive MPX infection in Europe:**	
Regarding its SEVERITY	(1) Not significant at all (2) (3) (4) (5) very significant
Regarding its FREQUENCY	(1) Not significant at all (2) (3) (4)<br>(5) very significant

**How would you rate the health threat represented by**	
Monkeypox Virus	(1) (2) (3) (4) (5) (6) (7) (8) (9) (10)
SARS-CoV-2	(1) (2) (3) (4) (5) (6) (7) (8) (9) (10)
Tuberculosis	(1) (2) (3) (4) (5) (6) (7) (8) (9) (10)
Seasonal influenza	(1) (2) (3) (4) (5) (6) (7) (8) (9) (10)
Hepatitis B Virus	(1) (2) (3) (4) (5) (6) (7) (8) (9) (10)
HIV	(1) (2) (3) (4) (5) (6) (7) (8) (9) (10)

**From your point of view, in the following 12 months Monkeypox**	
1)… will be a likely occurrence during daily activities	
Totally disagree	[ ]
Disagree	[ ]
Neutral	[ ]
Agree	[ ]
Totally Agree	[ ]
2)… will significantly affect your daily activities	
Totally disagree	[ ]
Disagree	[ ]
Neutral	[ ]
Agree	[ ]
Totally Agree	[ ]
**Are you confident to be able to recognize incident Monkeypox cases during your daily activities?**	
Totally disagree	[ ]
Disagree	[ ]
Neutral	[ ]
Agree	[ ]
Totally Agree	[ ]

Section 5. Some personal informations about you:.

Year of birth: ____________ Year of graduation: ____________

Gender: M [ ] F [ ] Prefer not to answer [ ]

Region of residence:

[ ] Northern Italy	Aosta Valley, Piedmont, Liguria, Lombardy, Veneto, Autonomous Province of Trento, Autonomous Province of Bolzano, Friuli-Venezia-Giulia, Emilia Romagna
[ ] Central Italy	Tuscany, Umbria, Marche, Lazio
[ ] Southern Italy / Islands	Campania, Abruzzo, Apulia, Basilicata, Calabria, Sicily, Sardinia
[ ] Other countries	

At the moment, you are employed as:
[ ] Occupational Physician
[ ] General Practitioner
[ ] Public Health Professional
[ ] Other medical Specialty (internal medicine)
[ ] Other medical Specialty (surgery)
[ ] Other medical Specialty (diagnostic or laboratory)
[ ] Other medical Specialty (dermatology)
[ ] Other medical Specialty (ICU)
[ ] Other medical Specialty (Pediatrics)
[ ] Other ______________________________
